# Experience and side effects of COVID-19 vaccine uptake among university students: a cross-sectional survey study

**DOI:** 10.3389/fpubh.2024.1361374

**Published:** 2024-06-24

**Authors:** Md. Akhtarul Islam, Mst. Tanmin Nahar, Abdur Rahman, A. S. M. Monjur Al Hossain, Umme Johra Jui, Tarana Tabassum, Sutapa Dey Barna, Shafia Tahmida, Afrina Akter Mishu, Shahanaj Parvin, Jannatul Naime, Razaz Waheeb Attar, Renad Waheeb Attar, Md. Tanvir Hossain

**Affiliations:** ^1^Statistics Discipline, Science Engineering & Technology School, Khulna University, Khulna, Bangladesh; ^2^Department of Pharmaceutical Technology, Faculty of Pharmacy, University of Dhaka, Dhaka, Bangladesh; ^3^Department of Bangla, University of Dhaka, Dhaka, Bangladesh; ^4^Department of Statistics, Comilla University, Cumilla, Bangladesh; ^5^Department of Statistics, Jagannath University, Dhaka, Bangladesh; ^6^Management Department, College of Business Administration, Princess Nourah bint Abdulrahman University, Riyadh, Saudi Arabia; ^7^Department of Medical Education, College of Medicine, King Abdulaziz University, Jeddah, Saudi Arabia; ^8^Sociology Discipline, Social Science School, Khulna University, Khulna, Bangladesh

**Keywords:** COVID-19, vaccine, experience, side effects, university students, Bangladesh

## Abstract

**Introduction:**

Many people expressed concern over coronavirus vaccinations’ reliability and side effects. This research aimed to assess university students’ perceptions and experiences regarding the side effects of the COVID-19 vaccines in Bangladesh.

**Method:**

We conducted an online cross-sectional survey to collect responses from university students vaccinated with any vaccines administered in Bangladesh between November 2021 to April 2022. Bangladeshi university students over the age of 18 and having an internet connection was included in the study. A binary logistic regression analysis along with Pearson’s Chi-square test were used to identify COVID-19 vaccine-related side effects predictors after receiving the first dose.

**Results:**

A total of 1,176 participants responded voluntarily to the online study, and most were vaccinated. More than half of the participants received the Sinopharm vaccine (56.5%), while others received Covishield (8.9%), Moderna (7.3%), and Pfizer (5.8%) vaccine. Around 32% of the participants reported side effects after receiving the first dose of the vaccine, including pain and edema (78.4%), body temperature (20.3%), and headache (14.5%), while a few experienced allergy, anxiety, and uneasy feelings. About 17% of the participants reported experiencing side effects after the second dose of the vaccine, including pain and edema (7.5%), body temperature (8.8%), and headache (7.3%). Most side effects were significantly associated with the Moderna vaccine (*p* < 0.001). Female students and those previously infected with COVID-19 were significantly associated with the side effects after taking the first dose of the vaccine.

**Conclusion:**

We found that side effects are mild and did not pose a significant challenge to Bangladesh’s effort in managing and reducing the risk associated with the COVID-19 pandemic.

## 1 Introduction

The dreadful spread of SARS-CoV-2 virus with its several arbitrarily mutated variants has been a severe global health concern due to the horrendous catastrophic effects on every sector of life across the world (1, 2). Following the declaration of the COVID-19 outbreak as a pandemic on March 11, 2020 by WHO, an ample amount of confirmed cases of the infection and deaths are being reported throughout the world till now (3). As of March 03, 2024, the number of reported confirmed cases is over 77,48,34,251, including 70,37,007 deaths (4). The abundance of the infection is higher in Europe and the Americas than in the Western Pacific and South-East Asia, Eastern Mediterranean, and Africa (4). In Bangladesh, the very first three cases of the SARS CoV-2 infection were confirmed and reported on March 08 2020, by the ‘Institute of Epidemiology, Disease Control and Research (IEDCR)’ (5, 6). Since then, both the rates of infection and death have been increasing across the country. As of March 03 2024, the nationwide numerical value of COVID-19 confirmed cases is 20,48,588, including a total of 29,491 deaths (4).

As a curative measure, several existing allopathic drugs, herbal medicines, and bioactive phytoconstituents found to be somewhat functional against coronavirus disease (2, 7); but yet to achieve herd immunity against COVID-19 vaccines are considered as promising preventive candidates. Because vaccines have been found to be effective against viral infections earlier (8). Therefore, on an emergency basis, to eliminate the spread and catastrophic effects of the novel strains of SARS CoV-2 virus around the world, several vaccines have been licensed worldwide with a short period of development stage (9); including Pfizer (96% efficacy rate), Moderna (94.10% efficacy rate), AstraZeneca-Oxford (70% efficacy rate) and Russian Sputnik V (92% efficacy rate) (10). As of November 26 2023, a total of 13.59 billion doses of different vaccines have been administered worldwide (4). While the Bangladeshi government commenced its nationwide mass vaccination program on February 07, 2021, prioritizing the frontline personnel, doctors, nurses, and older adult people on an initial basis, which is currently applicable for anybody over 18 years old with National Identity Card (10). Notably, the government also brought a massive number of students under vaccination coverage to resume the regular educational scheme after an extensive lockdown phase, which was imposed in the earlier days of the pandemic (10). As of November 26, 2023, in Bangladesh, a total of 15,15,04,394 vaccine doses have been administered nationwide (4); which include five different vaccines, namely, AstraZeneca (Covishield), Pfizer-BioNTech, Sinopharm, Moderna and Sinovac (Coronavac) (4).

Bangladesh began an extensive mass vaccination campaign for anyone above 18 with a national identity to deliver immunizations to university students (11). Despite significant advances in vaccination rollouts, vaccine hesitancy remains a key concern and a barrier to mass immunization success (12, 13). Vaccine reluctance and acceptance are multifaceted, and vaccine responses might differ depending on context, time, vaccine type, and geographical location (14). Despite the availability of vaccination services, vaccine hesitancy is defined as a delay in accepting or avoiding immunization (15). Hence, the precise causes of vaccination apprehension are unknown (16). However, the rate of acceptance or reluctance to take COVID-19 vaccination among Bangladeshis is different from that of other countries due to a lack of public awareness about the efficacy of these novel vaccines, the availability of several different manufacturer’s vaccines, the spread of controversial news in media, mistrust, misconception and cultural, religious, gender or other contemporary socioeconomic factors (17, 18).

For the implementation and estimation of the effectiveness of a nationwide mass vaccination campaign, we need to analyze and study the knowledge, thoughts, attitudes, responses, and opinions of the country’s common people about vaccination (19). Though several considerable survey researches regarding progression and perceptions of pre-and post-vaccination programs at times have been conducted on diversified common Bangladeshi people (11, 17, 20), surveys including the resident and non-resident university students can hardly be found. In addition, the public health sector demands more permutated survey studies in order to ensure broader community uptake of vaccines.

Therefore, this study’s core and primary objective was to explore the behavioral measurements and perceptions regarding COVID-19 vaccination of Bangladeshi undergraduate and post-graduate students of different ages, genders, religions, regions, and lifestyles from different universities across the country. The secondary objective of the study was to estimate the possible side effects of vaccination among the participants along with its relationship with the participant’s gender. Hopefully, the findings of this study will help the country’s respective legislators correlate the public health sector with the education sector.

## 2 Methods

### 2.1 Study design and participants

After Bangladesh launched the COVID-19 vaccination programs, a cross-sectional online survey of university students was conducted from November 2021 to April 2022 in order to portray the actual scenario regarding the perception and experience of the COVID-19 vaccination. This online survey was conducted irrespective of location and type (general or technical) of universities across Bangladesh, where the participants were selected using convenience sampling based on some specifications: (i) s/he must have access to the internet; (ii) s/he must be enrolled in the regular programs (undergraduate and postgraduate programs) universities of Bangladesh; (iii) s/he must be over 18 years of age; and (iv) s/he must be eligible for the vaccination program initiated by the universities. Considering the aforementioned criteria, university students were approached through social media (e.g., Facebook, Messenger, Instagram, WhatsApp, LinkedIn), and a total of 1,176 valid responses were retained in this study.

### 2.2 E-questionnaire

The e-questionnaire used in this online-based cross-sectional study was developed based on the existing literature (9, 11, 21, 22), and the e-questionnaire was in English as the medium of instruction in tertiary education in Bangladesh is English. The e-questionnaire has five modules; Section I extracted socio-demographic information, including age, religion, sex, residence, marital status, living arrangement, and household composition; Section II focused on the information of their respective educational institutions, such as the name of the university, level of education, and department/discipline; Section III highlighted the health status of the participants, including presence or absence of chronic diseases; while Section IV and section V contained questions regarding vaccination and their experience and opinion regarding the COVID-19 vaccine, respectively. The survey inquired about participants’ experiences and opinions regarding COVID-19 vaccination, including level of side effects (if any), physical or mental illness and types of such illness, medication usage to reduce the side effects, concerns about receiving the second dose, post-vaccination symptoms, hospitalization, recovery duration, and post-recovery difficulties.

### 2.3 Consent and ethical considerations

The institutional ethical clearance committee has approved this study (Protocol Number – KUECC-2021/11/32). In this study, the participants participated voluntarily after filling out a written informed consent form that assured their anonymity and the confidentiality of information. Moreover, the participants were free to decline the online survey without providing any prior explanation.

### 2.4 Statistical analysis

Data were analyzed using IBM SPSS Statistics (version 26.0) and Microsoft Excel 2019. After closing the online survey, the data were cleaned, sorted, edited – where necessary, and coded in Microsoft Excel. Later, the Excel file was imported into SPSS. First-order analysis, i.e., descriptive statistics and Pearson’s Chi-square (χ2), were used to describe the participants’ basic characteristics and identify their association with COVID-19 vaccine-related side effects. A binary logistic regression analysis was conducted to identify COVID-19 vaccine-related side effects predictors after receiving the first dose. The results included the odds ratio (OR) at a 95% confidence interval (CI). A *p*-value of 0.05 or less was used to establish what constitutes a statistically significant difference or association between the test populations.

## 3 Patient and public involvement

No patients were involved in this study.

## 4 Results

Among the participants, the majority fall in the age group of 22 to 24 years, which is 52%. Others fall into the age group of 18 to 21 years (36.4%), and rest of the participants are older than 25 years old (11.6%). 59% of respondents are male, while the remaining 41% are female. Islam is the most widely followed religion (86.9%), and 13.1% of respondents are from other religions. People partaking in the survey are mostly living with their families (55.9%), and 44.1% of the participants have made their living arrangements on their own. Most of the participants (87.7%) are either undergrads or graduates, and the rest have postgraduates’ degrees or other qualifications. Table 1 presents additional significant demographic characteristics of respondents. Fifty-six percent of the respondents have a good history of health, 35.2% have suffered from allergy diseases, and very few people had asthma, diabetes, heart disease, hypertension, or tuberculosis. 71.6% of the contributors did not test for COVID-19 as there were no symptoms, whereas 13.5% of the respondents did not test but had all the symptoms. 9.6% and 5.3% of people tested negative and positive for COVID-19, respectively. As most of the people did not test for COVID-19, the recovery period is not applicable to them, but 5.4% of participants have taken 1 to 3 days to fully recover, and 2% of people have taken more than 3 days to recover. 84.4% of the respondents have taken at least one dose of the vaccine; 226 completed the first dose, and 756 took both doses. Most of the participants who have taken the vaccine have taken Sinopharm, which is 56.5%. 63.4% of the total participants did not maintain at least 1.5 meters of social distancing, whereas the rest of the 36.6% maintained it. The majority of the respondents (85.5%) have continued to wear a face mask or shield. 73.5% of the participants have not continued washing or sanitizing their hands. 63.4% of the respondents covered their noses and mouths while coughing and sneezing. It is observed that 68.1% of the respondents did not experience any physical or mental illness after taking the first dose of the vaccine, and the remainder have faced either one or more illnesses.

**Table 1:** Baseline characteristics of different selected variables.

Variable	Level	*N*	%
Socio-demographic information			
*Age (in years)*			
	18–21	428	36.4
	22–24	611	52.0
	25<	137	11.6
*Sex*			
	Male	694	59.0
	Female	482	41.0
*Religion*			
	Islam	1,022	86.9
	Others	154	13.1
*Living arrangement*			
	Alone	519	44.1
	With family	657	55.9
*Education*			
	Undergraduate	1,031	87.7
	Postgraduates and others	145	12.3
*Division*			
	Khulna	276	23.5
	Dhaka	318	27.0
	Rangpur	193	16.4
	Chittagong	165	14.0
	Barishal	35	3.0
	Mymensingh	59	5.0
	Rajshahi	120	10.2
	Sylhet	10	0.9
*Residence*			
	Rural	274	23.3
	Sub-urban	239	20.3
	Urban	663	56.4
*Marital status*			
	Unmarried	1,079	91.8
	Married	96	8.2
	Divorced	1	0.1
Health status during COVID-19			
*Health status*			
	Poor	134	11.4
	Good	658	56.0
	Excellent	384	32.7
*Diseases*			
	Allergy diseases	414	35.2
	Asthma diseases	69	5.9
	Diabetes diseases	6	0.5
	Heart diseases	6	0.5
	Hypertension diseases	41	3.5
	Tuberculosis diseases	1	0.1
*COVID-19 infection*			
	Tested negative	113	9.6
	Tested positive	62	5.3
	I did not test, but I had all the symptoms	159	13.5
	I did not test, because I did not have any symptoms	842	71.6
*Recovery from infection*			
	Not applicable	1,088	92.5
	1–3 days	64	5.4
	Above 3 days	24	2.0
*Preventive measures among people in the last 7 days*			
	Never	514	43.7
	Once	187	15.9
	Always	475	40.4
COVID-19 vaccination			
*Vaccination status*			
	Yes	992	84.4
	No	184	15.6
*First dose taken*			
	Yes	226	19.2
	No	950	80.8
*Second dose taken*			
	Yes	756	64.3
	No	420	35.7
*Vaccine*			
	Moderna	86	7.3
	Covisheild	105	8.9
	Sinopharm	665	56.5
	Pfizer–BioNTech	68	5.8
	Sinovac (Coronavac)	21	1.8
	Sputnik V	2	0.2
	Janssen (Johnson & Johnson)	1	0.1
	Not Applicable	228	19.4
*Social distancing in public spaces*			
	Yes	430	36.6
	No	746	63.4
*Wearing face mask/shield*			
	Yes	1,005	85.5
	No	171	14.5
*Washing or sanitizing hands*			
	Yes	312	26.5
	No	864	73.5
*Covering mouth and nose when coughing and sneezing*			
	Yes	746	63.4
	No	430	36.6
*Physical or mental illness after taking the first dose of the vaccine*			
	Yes	375	31.9
	No	801	68.1
Physical or mental illness after 1st dose			
*Pain and edema*			
	Yes	254	21.6
	No	922	78.4
*Headache*			
	Yes	170	14.5
	No	1,006	85.5
*Increased body temperature*			
	Yes	239	20.3
	No	937	79.7
*Loss of appetite*			
	Yes	19	1.6
	No	1,157	98.4
*Allergy*			
	Yes	25	2.1
	No	1,151	97.9
*Rash/Itching*			
	Yes	15	1.3
	No	1,161	98.7
*Imbalance/lethargic*			
	Yes	9	0.8
	No	1,167	99.2
*High blood pressure*			
	Yes	8	0.7
	No	1,168	99.3
*Low blood pressure*			
	Yes	15	1.3
	No	1,161	98.7
*Anxiety*			
	Yes	27	2.3
	No	1,149	97.7
*Uneasy feeling/tension*			
	Yes	62	5.3
	No	1,114	94.7
*Pain and edema*			
	Yes	254	21.6
	No	922	78.4
*Medicine taken after the side effects of the first dose*			
	Yes	250	21.3
	No	926	78.7
Medicine after first dose			
*Painkiller*			
	Yes	92	7.8
	No	1,084	92.2
*Sedative*			
	Yes	5	0.4
	No	1,171	99.6
*Anti-allergic medicine*			
	Yes	13	1.1
	No	1,163	98.9
*Fever medicine*			
	Yes	183	15.6
	No	993	84.4
*Medications for abnormal high blood pressure*			
	Yes	3	0.3
	No	1,173	99.7
Physical or mental illness after second dose			
*Physical or mental illness after taking the second dose*			
	Yes	194	16.5
	No	631	53.7
*Pain and edema*			
	Yes	88	7.5
	No	1,088	92.5
*Headache*			
	Yes	86	7.3
	No	1,090	92.7
*Increased body temperature*			
	Yes	104	8.8
	No	1,072	91.2
*Loss of appetite*			
	Yes	1,157	98.4
	No	19	1.6
*Allergy*			
	Yes	20	1.7
	No	1,156	98.3
*Anxiety*			
	Yes	8	0.7
	No	1,168	99.3
*Imbalance/lethargic*			
	Yes	2	0.2
	No	1,174	99.8
*Rash/Itching*			
	Yes	9	0.8
	No	1,167	99.2
*Low BP*			
	Yes	4	0.3
	No	1,172	99.7
Medicine second dose			
*Fever medicine*			
	Yes	95	8.1
	No	1,081	91.9
*Painkiller*			
	Yes	42	3.6
	No	1,134	96.4
*Anti-allergic medicine*			
	Yes	4	0.3
	No	1,172	99.7
*Admitted it the hospital or ICU after taking the vaccine*			
	Yes	13	1.1
	No	1,163	98.9

After the first dose of the vaccine, there was no experience of pain and edema (78.4%), headache (85.5%), or increased body temperature (79.7%). There was no loss of appetite (98.4%) or allergy (97.9%). Few people have experienced allergy symptoms after taking the first dose.

Among other medical statuses, there was no experience of rash or itching on the skin (98.7%), no imbalance or lethargic behavior (99.2%), no high blood pressure (99.3%), and no low blood pressure problem (98.7%). Among the mental conditions, 97.7% did not feel any anxiety, and 94.7% had no uneasy feelings or tension.

As a result of all these medical conditions, 78.7% of the participants did not take any medicine for the side effects after taking the first dose of the vaccine, and 21.3% of the respondents have taken either one or more medicines to mitigate the side effects. Most (92.2%) of the participants have not taken any painkiller, and almost everyone (99.6%) has not taken any sedative after taking the first dose of the vaccine. No use of anti-allergic medicine (98.9%), fever medicine (84.4%), or high blood pressure medicine (99.7%) was seen among the respondents.

16.5% of the participants reported at least one kind of mental or physical illness, but the majority reported no such problems (53.7%) after taking the second dose of the vaccine. Among medical conditions after taking the second dose of the vaccine, no pain and edema (92.5%), no headache (92.7%), no rise in body temperature (91.2%), no loss of appetite (99.2%), and no anxiety (99.3%) were seen among the participants. Almost everyone who took the second medicine reported no rash, itching (99.2%), or abnormally low blood pressure (99.7%). As a result, the no fever medicine (91.9%) and no painkiller and anti-allergic medicine (98.9%) intake was very high after the second dose. The vast majority (98.9%) reported no admission to the hospital or I.C.U. after taking any dose of the vaccine after being infected with COVID-19.

Table 2 shows a significant difference in side effects between male and female participants with a previous history of COVID-19 infection after each dose of the vaccine. Analysis shows that female participants experience side effects more than male participants in both the first and second doses. There was a significant difference in the number of patients reporting pain and edema (18.6% male and 25.9% female) after receiving the first and second doses of the vaccine (males 5.2% and females 10.8%). Moreover, the result shows a significant (*p* = 0.016) difference in the headache for gender (males 12.4% and females 17.4%) in the first dose but no significant (*p* = 0.279) difference after the second dose. There is a significant association between gender and increased body temperature (*p* = 0.008 for the first dose and *p* = 0.005 for the second dose) after each dose of the vaccine. In both cases, increased body temperature is reported mostly by female participants. It is also seen that there is a significant (*p* = 0.003) symptom of appetite loss from males (0.7%) to females (2.9%) in the first dose, although there is no significant (*p* = 0.372) difference between males (0.6%) and females (1.0%) after the second dose.

**Table 2:** Gender-based comparison of side effects between the 1st and 2nd doses of the COVID-19 vaccine.

**Experience and symptoms**	**After 1st dose of COVID-19 vaccine frequency (%)**			**After 2nd dose of COVID-19 vaccine frequency (%)**		
	**Male *N* = 684**	**Female *N* = 482**	***p*-value**	**Female *N* = 482**	**Male *N* = 684**	***p*-value**
Experience of side effects after COVID-19 vaccination						
No	502 (72.3)	299 (62.0)	<0.001	400 (57.6)	231 (47.9)	<0.001
Yes	192 (27.7)	183 (38.0)		91 (13.1)	103 (21.4)	
Symptoms						
Pain and Edema	129 (18.6)	125 (25.9)	0.003	36 (5.2)	52 (10.8)	<0.001
Headache	86 (12.4)	84 (17.4)	0.016	46 (6.6)	40 (8.3)	0.279
Increased body temperature	123 (17.7)	116 (24.1)	0.008	48 (6.9)	56 (11.6)	0.005
Loss of appetite	5 (0.7)	14 (2.9)	0.003	4 (0.6)	5 (1.0)	0.372
Allergy	11 (1.6)	14 (2.9)	0.123	9 (1.3)	11 (2.3)	0.199
Rash/Itching	4 (0.6)	11 (2.3)	0.010	2 (0.3)	7 (1.5)	0.024
Imbalance/Lethargic	4 (0.6)	5 (1.0)	0.372	1 (0.1)	1 (0.2)	0.795
High blood pressure	5 (0.7)	3 (0.6)	0.841	2 (0.3)	2 (0.4)	0.713
Low blood pressure	6 (0.9)	9 (1.9)	0.132	2 (0.3)	2 (0.4)	0.713
Anxiety	17 (2.4)	10 (2.1)	0.673	6 (0.9)	2 (0.4)	0.356
Uneasy feeling/tension	36 (5.2)	26 (5.4)	0.876	0 (0.0)	0 (0.0)	<0.001

Rash or itching showed a significant association after the first and second doses. Furthermore, in the case of allergy, imbalance, high blood pressure, low blood pressure, anxiety, or an uneasy feeling, there is no significant difference between males and females for either of the doses.

Table 3 presents the students’ information regarding the side effects of different types of vaccines. It is visible that both doses of the Moderna vaccine are significantly associated with the reporting of pain and edema and increased body temperature symptoms compared to the Covishield, Pfizer, Sinopharm, and Sinovac vaccines. More than half of the participants receiving the Moderna vaccine have reported pain, edema, and increased body temperature after the vaccination. Other vaccines, such as Covishield, Sinopharm, and Pfizer, showed mild side effects after vaccination.

**Table 3:** Vaccine-wise side effects after each dose of the COVID-19 vaccine.

**Symptoms**	**After 1st dose of the COVID-19 vaccine** frequency (%)						**After 2nd dose of the COVID-19 vaccine** frequency (%)					
	**Moderna**	**Covishield**	**Sinopharm**	**Pfizer**	**Sinovac**	***p*-value**	**Moderna**	**Covishield**	**Sinopharm**	**Pfizer**	**Sinovac**	***p*-value**
Pain and Edema	48 (55.8)	42 (40.0)	114 (17.1)	25 (36.8)	4 (19.0)	<0.001	27 (31.4)	10 (9.5)	48 (7.2)	2 (2.9)	0 (0.0)	<0.001
Headache	30 (34.9)	36 (34.3)	73 (11.0)	12 (17.6)	1 (4.8)	<0.001	24 (27.9)	13 (12.4)	39 (5.9)	5 (7.4)	0 (0.0)	<0.001
Increased body temperature	53 (61.6)	61 (58.1)	80 (12.0)	19 (27.9)	4 (19.0)	<0.001	46 (53.5)	16 (15.2)	33 (5.0)	6 (8.8)	2 (9.5)	<0.001
Loss of appetite	3 (3.5)	8 (7.6)	4 (0.6)	2 (2.9)	0 (0.0)	<0.001	3 (3.5)	2 (1.9)	3 (0.5)	1 (1.5)	0 (0.0)	0.062
Allergy	6 (7.0)	2 (1.9)	14 (2.1)	2 (2.9)	0 (0.0)	0.059	5 (5.8)	0 (0.0)	14 (2.1)	1 (1.5)	0 (0.0)	0.030
Rash/Itching	4 (4.7)	2 (1.9)	7 (1.1)	1 (1.5)	0 (0.0)	0.190	1 (1.2)	0 (0.0)	6 (0.9)	1 (1.5)	0 (0.0)	0.954
Imbalance/Lethargic	1 (1.2)	3 (2.9)	3 (0.5)	1 (1.5)	0 (0.0)	0.329	1 (1.2)	0 (0.0)	0 (0.0)	1 (1.5)	0 (0.0)	0.061
High blood pressure	1 (1.2)	1 (1.0)	4 (0.6)	0 (0.0)	1 (4.8)	0.501	1 (1.2)	1 (1.0)	2 (0.3)	0 (0.0)	0 (0.0)	0.780
Low blood pressure	2 (2.3)	3 (2.9)	8 (1.2)	2 (2.9)	0 (0.0)	0.367	1 (1.2)	1 (1.0)	1 (0.2)	1 (1.5)	0 (0.0)	0.428
Anxiety	4 (4.7)	6 (5.7)	13 (2.0)	1 (1.5)	0 (0.0)	0.207	3 (3.5)	-	5 (0.8)	-	-	0.072
Uneasy feeling/tension	9 (10.5)	9 (8.6)	35 (5.3)	2 (2.9)	3 (14.3)	0.018	-	-	-	-	-	-

Table 4 represents the binary logistic predictors of COVID-19 vaccine side effects after the first dose. In the binary logistic regression model, the age group 22–24 has 1.335 times (aOR: 1.335, 95% CI: 1.012–1.763) higher odds of experiencing the side effect of COVID-19 vaccine than the age group 18–21. Female students are significantly 11.6% (aOR: 1.116, 95% CI: 0.846–1.472) more likely to experience any side effect of the COVID-19 vaccine than males. The table shows that students living in urban areas have 1.471 times (aOR: 1.471, 95% CI: 1.059–2.045) higher odds of experiencing side effects after the first dose than rural-based students.

**Table 4:** Binary logistic estimates for side effects of the first dose of the vaccine.

Variable	aOR	*B*	*p*-value	95% CI for EXP(B)	
				Lower	**Upper**
Age (in years)					
18-21^(r)^					
22–24	1.335	0.289	0.041	1.012	1.763
25<	1.247	0.220	0.382	0.760	2.044
Sex					
Male^(r)^					
Female	1.116	0.110	0.038	0.846	1.472
Residence					
Rural^(r)^					
Sub-urban	1.510	0.412	0.038	1.023	2.231
Urban	1.471	0.386	0.022	1.059	2.045
Living status					
Alone^(r)^					
With family	1.177	0.163	0.220	0.907	1.528
Education					
Undergraduate^(r)^					
Postgraduates and others	1.565	0.448	0.046	1.009	2.428
COVID-19 Status					
Negative^(r)^					
Positive	1.613	0.478	0.003	1.178	2.207
Heath status					
Poor^(r)^					
Good	0.810	−0.210	0.299	0.545	1.205
Excellent	0.761	−0.273	0.214	0.494	1.171
Recovery					
Not applicable^(r)^					
1–3 days	4.615	1.529	<0.001	2.642	8.060
Above 3 days	4.172	1.428	0.002	1.722	10.107
Allergy					
No^(r)^					
Yes	1.327	0.283	0.035	1.020	1.726
Social Distance					
No^(r)^					
Yes	0.669	−0.402	0.006	0.501	0.893
Covering mouth					
No^(r)^					
Yes	1.557	0.443	0.003	1.158	2.094

^r^ Indicates reference category; ^aOR^ Adjusted odds ratio.

Also, students living in suburban areas have 1.510 times (aOR: 1.510, 95% CI: 1.023–2.231) higher odds of experiencing side effects than rural respondents. Living arrangements have no impact on the side effects of the COVID-19 vaccine since they are statistically insignificant.

Table 4 also shows that postgraduate students are 1.565 times (aOR: 1.565, 95% CI: 1.009–2.428) more likely to experience the side effect of the COVID-19 vaccine than undergraduate students, with a *p*-value of 0.046. Health status and living arrangements have no impact on the side effects of the COVID-19 vaccine since the findings are not statistically significant.

Compared to students who did not contract SARS-CoV-2, those who did are 1.613 times (aOR: 1.613, CI: 1.178 to 2.207) more likely to experience any side effects following the initial dose. The recovery period is a significant factor; those students who needed 1–3 days to recover are 4.615 times (aOR: 4.615, 95% CI: 2.642–8.060) more likely to experience side effects after the first dose. Students who require more than 3 days to recover from COVID-19 are 4.172 times (aOR: 4.172, 95% CI: 1.722–10.107) more likely to experience side effects (*p*-value = 0.002). Allergy is a significant factor for side effects; respondents with allergies are 1.327 times (aOR: 1.327, 95% CI: 1.020–1.726) more likely to experience side effects after the first dose. Those who maintain a social distance of at least 15 meters in public spaces are 0.669 times (aOR: 0.669, 95% CI: 0.501–0.893) less likely, or 33.1% less likely, to experience any side effect of the COVID-19 vaccine after the first dose. Covering the mouth and nose when coughing and sneezing made people 1.557 times (aOR: 1.557, 95% CI: 1.158–2.094) more likely to experience side effects (*p* = 0.003).

## 5 Discussion

This study aimed to assess the perceptions and experiences of university students regarding the side effects of the COVID-19 vaccines in Bangladesh. The participants experienced different physical and mental illnesses after receiving the doses of the COVID-19 vaccine. This study shows that four out of five (84.4%) participants have taken at least one dose of any of the COVID-19 vaccines available for university students in Bangladesh. Some participants experienced pain and edema, growing body temperature, and headache after receiving the first dose, while very few reported uneasy feelings and tension after receiving the first dose. Likewise, after receiving the second dose, a few participants encountered body temperature, pain, edema, and headache. A similar result was found in a study in Bangladesh that observed a wide range of adverse side effects, such as fever, headache, and pain, where the vaccine was injected, which aligns with our study (23). Some other research revealed that the side effects caused by the COVID-19 vaccines are pain where the vaccine was injected, muscle pain, joint pain, fever, headache, tiredness, and fatigue (24–26), thus complementing the findings of our study. Usually a non-serious adverse event like pain, headache, and injection site pain are reported for every million doses administered (27). Injection site pain, headache, or fatigue was also common for 50% of participants in the clinical trial (28). The reported adverse effects after the first dose were non-serious and temporary in Bangladesh (9). From our study, it is also evident that the participant’s sex was substantially associated with the side effects of the COVID-19 vaccine after receiving both doses. Female participants were more likely to suffer from the general side effects than their male counterparts, which resembles previous studies (24, 29–33). Still, some contradictory results were found where no sex difference regarding the effects of the COVID-19 vaccine was reported among the vaccine recipients (34, 35). This may be due to information biases, as women are more likely to report their experiences than men (31). Besides, the difference between sex and endocrine hormones may play important roles in the higher report of side effects of the COVID-19 vaccine (36). Another reason may be that women are more susceptible to adverse events of pharmacokinetics and pharmacodynamics, or women may carry a significant percentage of body fat compared to men (37). The present study also suggested a significant relationship between different (symptoms) adverse effects and sex after receiving doses of the COVID-19 vaccine. For example, female students most likely suffered from pain, edema, and body temperature compared to male students. To the best of our knowledge, our study’s unique findings determined the association between sex and the specific type of adverse effects of COVID-19 vaccination.

This study uncovered another important aspect of COVID-19 vaccination, suggesting a significant association between vaccine types and side effects after vaccination (at least taking one of the doses). It was found that the majority of the participants received the Sinopharm vaccine, while others received Moderna, Covishield, and Pfizer-BioNTech vaccines. However it is seen that the first and second dose of the Moderna vaccine was significantly associated with higher reports of pain, edema, and body temperature symptoms compared to Covishield, Pfizer, Sinopharm, and Sinovac vaccines. More than half of the participants who received the Moderna vaccine reported feeling pain and body temperature after receiving it. This is similar to the findings of previous studies (32, 38–40). A possible reason for this outcome may be an inactive substance that serves as the vehicle or medium for other active agents utilized in the mRNA vaccine (Moderna). This agent serves as prevention against bacterial infections by invigorating a more grounded immune reaction, which may have significantly contributed to prompt reactions related to vaccination (41). Some of the other causes of these side effects may be due to a pre-existing physical problem (e.g., prior history of asthma, food or drug allergies), or it may be an unfortunate coincidence limited by medical science and has no relation with vaccination (42).

From binary logistic regression, some important predictors of side effects of the COVID-19 vaccine were identified. Female students were more likely to experience side effects of the COVID-19 vaccine than male students. Similar results were found in studies conducted in other countries, such as Saudi Arabia, Israel, the USA, Japan, and UAE (29–33, 36, 43). This study found that the participants with prior COVID-19 infection were more likely to experience side effects after taking the first dose. A cross-sectional study in the USA among healthcare workers with similar results acknowledged that the adverse reaction of the post-vaccination period was more prevalent among people who were infected by COVID-19 previously (42).

The age of the participants was another significant predictor of the possibility of experiencing adverse reactions after vaccination. Higher odds of side effects of the COVID-19 vaccine were found in the age group 22–24 compared to the age group 18–21, and this result is parallel with the findings of previous clinical trials in the Czech Republic, India, and Pakistan (37, 44, 45). The receptivity of the vaccine producing usual prospective adverse reactions declined with age as per these studies (37, 44, 45). However, it is not a possible cause of a desired resistant reaction (46).

We found inconsistent results demonstrating age as an independent predictor of the vaccine’s side effects (47). Another study claimed that they could not imbed a relationship between the vaccine’s adverse effects and age (48). A significant relationship was observed between the students having allergies and the side effects of the COVID-19 vaccine after the first dose. The participants with allergies were more likely to experience adverse effects after taking the first dose of the COVID-19 vaccine than those with no history of allergy. Some previous studies support this finding that the allergic history of the participants is usually the associated reason for adverse reactions to the COVID-19 vaccine (29, 38, 49, 50).

## 6 Strength and limitation

To the best of the authors’ knowledge, this is the first study to investigate the side effects of several COVID-19 vaccines among university students in Bangladesh. Previous studies on this particular topic focused mostly on healthcare workers and general people. Since university students are educated and adults, their responses recorded are mostly reliable. However, the study has several limitations too. Due to the convenience sampling approach, there might be some selection biases. Second, since the study was online, voluntary, and self-administered, we cannot confirm the seriousness of all participants while filling out the e-questionnaire, causing potential information bias. Third, the study participants are limited to university students aged 18–30, which may restrict generalization of the findings for other age groups. Fourth, the survey is online based so a group of students might miss the opportunity to be included in the survey due to lack of online facilities. Fifth, we do not have any information after the third, fourth or other consecutive doses of the vaccines, which might reflect a different scenario. Future studies should try to address these issues with more inclusive analysis and data collection strategies.

## 7 Conclusion

The COVID-19 vaccination effectively protects against fatal disease, hospitalization, and death. However, the recipients reported a wide range of side effects, which may not be severe or fatal. It is reported that side effects (pain and edema, body temperature, and headache) were reported mainly by recipients of Moderna, while recipients of other vaccines (Covishield, Sinopharm, and Pfizer) also showed side effects after vaccination, though they were mild (allergies, anxiety, and uneasy feelings). Side effects were mainly reported after the first dose of the COVID-19 vaccination. Side effects of the COVID-19 vaccine varied between sexes, previous infection status, and individuals with allergy issues. Finally, this study’s findings might help policymakers choose the vaccine for the mass population with minimal side effects.

## Data availability statement

The datasets presented in this study can be found in online repositories. The names of the repository/repositories and accession number(s) can be found at: https://dataverse.harvard.edu/dataset.xhtml?persistentId=doi:10.7910/DVN/NIIRDQ.

## Ethics statement

The studies involving humans were approved by Khulna University Ethical Clearance Committee (Protocol Number – KUECC-2021/11/32). The studies were conducted in accordance with the local legislation and institutional requirements. The participants provided their online informed consent to participate in this study. Online informed consent was obtained from the individual(s) for the publication of any potentially identifiable images or data included in this article. Moreover, the participants were free to decline the online survey without providing any prior explanation.

## Author contributions

MAI: Conceptualization, Data curation, Formal analysis, Investigation, Methodology, Software, Supervision, Writing – original draft, Writing – review & editing. MTN: Data curation, Formal analysis, Methodology, Software, Writing – original draft. AR: Investigation, Writing – original draft, Writing – review & editing. ASMMAH: Data curation, Investigation, Writing – original draft. UJJ: Data curation, Investigation, Writing – original draft. TT: Data curation, Investigation, Writing – original draft. SDB: Data curation, Investigation, Methodology, Writing – original draft. ST: Data curation, Investigation, Writing – original draft. AAM: Data curation, Investigation, Writing – original draft. SP: Data curation, Investigation, Writing – original draft. JN: Data curation, Investigation, Writing – original draft. RaWA: Writing – review & editing, Funding acquisition, Resources. ReWA: Resources, Writing – review & editing. MTH: Conceptualization, Validation, Writing – review & editing, Supervision.
